# Cardiac ^123^I-*m*IBG Imaging in Heart Failure

**DOI:** 10.3390/ph15060656

**Published:** 2022-05-25

**Authors:** Derk O. Verschure, Kenichi Nakajima, Hein J. Verberne

**Affiliations:** 1 Department of Radiology and Nuclear Medicine, Amsterdam University Medical Center, Location Amsterdam Medical Center, University of Amsterdam, Meibergdreef 9, 1105 AZ Amsterdam, The Netherlands; h.j.verberne@amsterdamumc.nl; 2 Department of Cardiology, Zaans Medical Center, Koningin Julianaplein 58, 1502 DV Zaandam, The Netherlands; 3 Department of Functional Imaging and Artificial Intelligence, Kanazawa University, Kanazawa 920-8641, Japan; nakajima@med.kanazawa-u.ac.jp

**Keywords:** chronic heart failure, innervation, ^123^I-*m*IBG scintigraphy, heart-to-mediastinum ratio

## Abstract

Cardiac sympathetic upregulation is one of the neurohormonal compensation mechanisms that play an important role in the pathogenesis of chronic heart failure (CHF). In the past decades, cardiac ^123^I-*m*IBG scintigraphy has been established as a feasible technique to evaluate the global and regional cardiac sympathetic innervation. Although cardiac ^123^I-*m*IBG imaging has been studied in many cardiac and neurological diseases, it has extensively been studied in ischemic and non-ischemic CHF. Therefore, this review will focus on the role of ^123^I-*m*IBG imaging in CHF. This non-invasive, widely available technique has been established to evaluate the prognosis in CHF. Standardization, especially among various combinations of gamma camera and collimator, is important for identifying appropriate thresholds for adequate risk stratification. Interestingly, in contrast to the linear relationship between ^123^I-*m*IBG-derived parameters and overall prognosis, there seems to be a “bell-shape” curve for ^123^I-*m*IBG-derived parameters in relation to ventricular arrhythmia or appropriate implantable cardioverter defibrillator (ICD) therapy in patients with ischemic CHF. In addition, there is a potential clinical role for cardiac ^123^I-*m*IBG imaging in optimizing patient selection for implantation of expensive devices such as ICD and cardiac resynchronization therapy (CRT). Based on cardiac ^123^I-*m*IBG data risk models and machine learning, models have been developed for appropriate risk assessment in CHF.

## 1. Introduction

Chronic heart failure (CHF) is a clinical syndrome with a growing incidence and prevalence. In addition to the activated renin–angiotensin–aldosterone system and natriuretic peptides, myocardial sympathetic innervation is increased in patients with CHF. Initially, these neurohormonal systems are able to compensate for the impaired myocardial function. However, the long-term activation of these compensation mechanisms has detrimental effects on myocardial structure and function resulting in heart failure (HF) progression.

**Figure 1:**
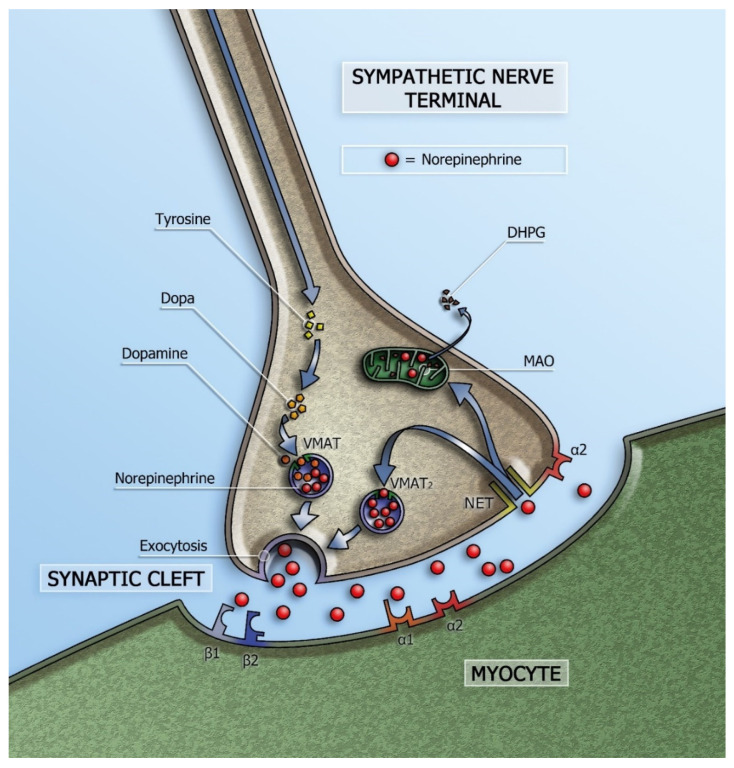
Schematic representation of the sympathetic synapse. Norepinephrine is synthesized within neurons by an enzymatic cascade. Dihydroxyphenylalanine (DOPA) is generated from tyrosine and subsequently converted to dopamine by DOPA decarboxylase. Dopamine is transported into storage vesicles by the energy-requiring vesicular monoamine transporter (VMAT). Norepinephrine is synthesized by dopamine β-hydroxylase within these vesicles. Neuronal stimulation leads to norepinephrine release through fusion of vesicles with the neuronal membrane (exocytosis). Apart from neuronal stimulation, release is also regulated by a number of presynaptic receptor systems, including α2–adrenergic receptors, which provide negative feedback for exocytosis. Most norepinephrine undergoes re-uptake into nerve terminals by the presynaptic norepinephrine transporter (NET) and is re-stored in vesicles (following uptake by vesicular amine transporter 2 (VMAT2)) or is metabolized in cytosol dihydroxyphenylglycol (DHPG) by monoamine oxidase (MAO). (Adapted from Verschure et al. [1].

Cardiac sympathetic innervation has several cardiovascular actions including heart rate acceleration (positive chronotropic effect) and increase in myocardial contractility (positive inotropic effect). Norepinephrine (NE) is the neurotransmitter of myocardial sympathetic innervation and is stored in vesicles in the presynaptic nerve terminals. Via exocytosis, NE is released into the synaptic cleft. Most of the released NE undergoes re-uptake into the presynaptic sympathetic terminal nerve axons via the uptake-1 mechanism. This transport system, the so-called norepinephrine transporter (NET), is responsible for approximately 70–90% of the NE re-uptake from the myocardial sympathetic synaptic cleft. As a consequence, only a small amount of the released NE will be available to stimulate the post-synaptic β-adrenergic receptors (β-AR) of the myocytes (Figure 1).

In CHF, the increased cardiac sympathetic activity is caused by an increased release of NE into the synaptic cleft. In addition, NE re-uptake via the NET is decreased resulting in elevated synaptic levels of NE. These elevated NE levels result in a spillover into the plasma with consequently increased plasma levels of NE concomitant with the severity of left ventricular dysfunction [2,3]. Initially, increased post-synaptic β-AR stimulation by increased NE levels helps to compensate for impaired myocardial function, but long-term NE excess has detrimental effects on myocardial structure and eventually leads to downregulation and decrease in sensitivity of post-synaptic β-AR with downstream effects on second messenger signaling (adenylate cyclase) [4,5]. This down regulation of post-synaptic β-AR causes left ventricular remodeling with further decrease in left ventricular ejection fraction (LVEF) and consequently increased morbidity and mortality.

Clinical assessment of myocardial sympathetic innervation can be performed by measuring NE plasma levels. Although increased NE plasma levels are associated with mortality in CHF [3], these levels do not specifically reflect the sympathetic activity at the cardiac level. Furthermore, these measurements of NE plasma levels are time consuming and there is a high variability in measurements. Other methods to measure cardiac sympathetic innervation are heart rate variability (HRV) using spectral analysis, muscle sympathetic nerve activity using microneurography and cardiac NE spill over using radiolabeled techniques. However, these techniques have limitations in qualitatively and quantitatively measuring selective cardiac sympathetic activation [6,7].

To date, cardiac sympathetic innervation can easily be visualized by non-invasive nuclear techniques. The most commonly used tracers are ^123^I-*meta*-iodobenzylguanidine (^123^I-*m*IBG) for planar and SPECT imaging and ^11^C-hydroxyephedrine (HED) for PET imaging. Both tracers are NE analogs resistant to metabolic enzymes and show high affinity for NET allowing the visualization of presynaptic sympathetic nerve function. Other presynaptic PET tracers include ^11^C-epinephrine, ^11^C-phenylephrine, and ^18^F-flubrobenguana [8,9]. Therefore, it is important to know that these PET tracers differ in their affinity to the NET, vesicular storage and metabolism, with consequently differences in their kinetics and specificity (Table 1). For clinical practice, the availability of tracers, is essential. Compared to ^11^C-HED which is labeled with a short–half-life isotope (20 min), ^123^I-*m*IBG can be centrally manufactured and then distributed. To overcome the issue of availability and distribution of PET tracers, an ^18^F-labeled compound for cardiac sympathetic innervation PET imaging has been developed [10]. Recently, a study demonstrated that the novel PET tracer ^18^F-Flubrobenguane (FBBG) yield equivalent global and regional distributions in both patients with and without ischemic CHF [11]. In addition, comparative studies between different imaging techniques for the assessment of cardiac sympathetic activity are currently lacking.

So, although the development of a ^18^F isotope for PET imaging is ongoing, for the near future ^123^I-*m*IBG scintigraphy will remain the most widely available planar/SPECT imaging method for assessing global and regional cardiac sympathetic innervation. Therefore, the focus of this review will be on cardiac ^123^I-*m*IBG imaging only.

## 2. Cardiac ^123^I-*m*IBG Imaging

Since its introduction, cardiac ^123^I-*m*IBG scintigraphy has been established as a highly reproducible and feasible technique to evaluate the global and regional cardiac sympathetic innervation [19,20,21]. Parameters of ^123^I-*m*IBG myocardial uptake and ^123^I-*m*IBG washout (WO) have been shown to be of clinical value, especially for the assessment of prognosis, in many cardiac and neurological diseases [22,23,24,25,26].

### 2.1. Patient Preparation

It has been known that some drugs may interfere with ^123^I-*m*IBG uptake [27]. However, many cardiac ^123^I-*m*IBG studies have conducted in CHF patients on optimal medical therapy (OMT), including beta-blockers, angiotensin-converting enzyme inhibitors (ACE-I) and angiotensin receptor blockers (ARB) [28,29]. Despite OMT, cardiac ^123^I-*m*IBG imaging is able to estimate the residual risk in these patients. So, there is no need to withdraw such medication prior to cardiac ^123^I-*m*IBG imaging. To prevent increase in thyroid activity over time because of uptake of free ^123^I subjects are pre-treated with 250 mg oral potassium iodide. However, in most subjects, there is still a low-level ^123^I-*m*IBG thyroid activity that probably represents specific uptake in the sympathetic nerve terminal in the thyroid [30].

### 2.2. Planar ^123^I-mIBG Acquisition and Analysis

**Figure 2:**
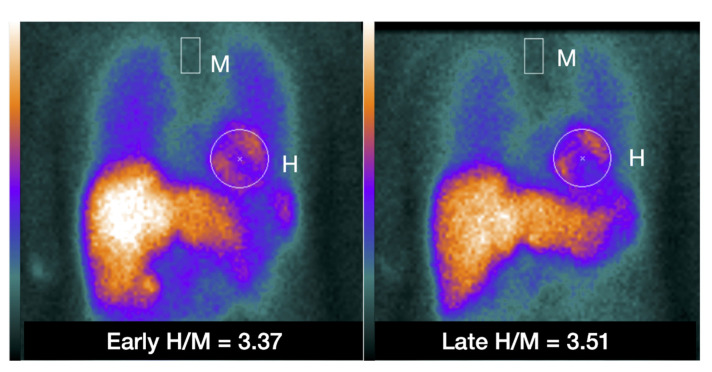
Example of placing a circular or elliptical region of interest (ROI) over the heart (H) and fixed rectangular mediastinal ROI placed on the upper part of the mediastinum (M) for calculating heart-to-mediastinum ratio (H/M). The same ROIs are placed on early and late images to calculate H/M and washout. The H/M outcomes are standardized to the ME-collimator condition.

**Figure 3:**
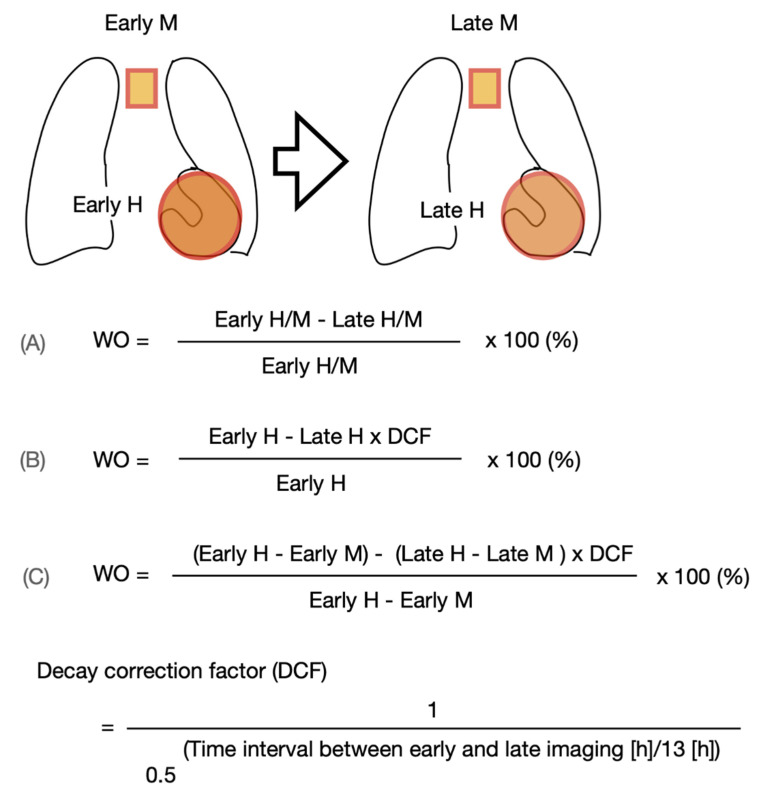
Variations of ^123^I-*m*IBG washout (WO) calculation using the myocardial count densities requiring a time decay correction factor (DCF) without (B) or with background correction (C). Calculating the DCF value by the formula of 1/0.5^(time/13)^ for 3.0, 3.5 or 4.0 h, the DCF are 1.17, 1.21 and 1.24, respectively.

Subjects will be injected with ^123^I-*m*IBG intravenously (111–370 MBq, depending on local use, regulations and gamma camera sensitivity). Fifteen minutes (early acquisition) and 4 h (late acquisition) after administration of ^123^I-*m*IBG, 10 min planar images are acquired with using a gamma camera equipped with a low energy high resolution (LEHR) or medium (ME) collimator. Recommended imaging acquisition conditions for cardiac ^123^I-*m*IBG imaging are shown in Table 2 The most widely semi-quantitative parameters in cardiological and neurological applications are the early and late heart-to-mediastinal ratio (H/M) and ^123^I-*m*IBG WO. A region-of-interest (ROI) is set as a cardiac contour, ellipsoid or circle over the heart (Figure 2). Standardized background correction is derived from a fixed rectangular mediastinal ROI (7 × 7 pixels) placed on the upper part of the mediastinum [27]. The location of the mediastinal ROI is determined in relation to the lung apex, the lower boundary of the upper part of the mediastinum, and the midline between the lungs. The H/M is determined by dividing the average counts (count/pixel) of the heart by the average count (count/pixel) of the mediastinum [27]. Figure 3 shows several formulas to calculate ^123^I-*m*IBG WO. The most commonly used calculation of ^123^I-*m*IBG WO is formula A using early and late H/M only. The early H/M offers predominantly information about the integrity of sympathetic nerve terminals (i.e., number of functioning nerve terminals and intact NET), while the late H/M offers information about the neuronal function resulting from uptake, storage and release. The ^123^I-*m*IBG WO reflects predominantly neuronal integrity of sympathetic tone/adrenergic drive [31].

### 2.3. SPECT ^123^I-mIBG Acquisition and Analysis

**Figure 4:**
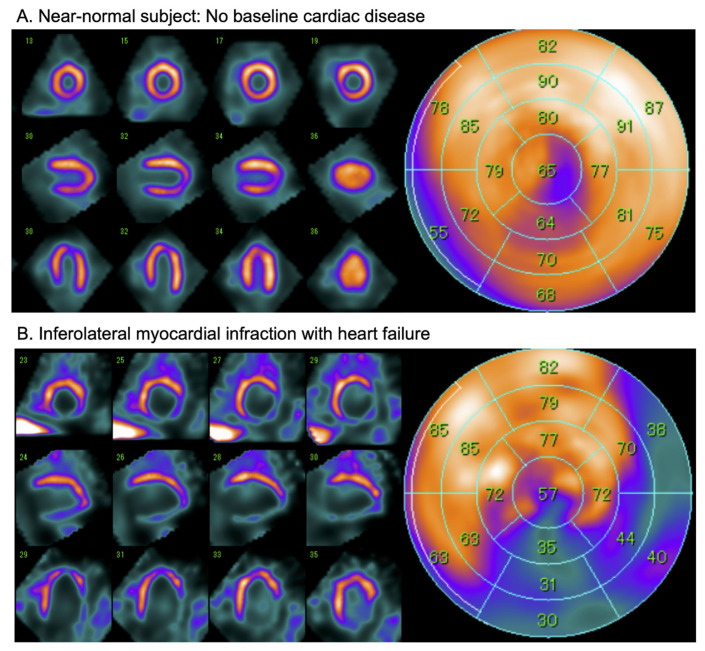
Examples of late ^123^I-*m*IBG CZT SPECT (D-SPECT, Spectrum Dynamics) imaging in a near-normal patient (**A**) and a patient with after an inferolateral myocardial infarction (**B**). Conventional short-axis, vertical and horizontal axis slices (left panel), and the corresponding 17-segment model polar map (right panel).

Compared with the H/M derived from two-dimensional planar images, three-dimensional imaging using single-photon emission tomography (SPECT) provides a more complete understanding of regional cardiac sympathetic innervation [32]. Although an officially established method for scoring ^123^I-*m*IBG SPECT images is lacking, analysis can be performed similar to conventional 17-segment/5-point model used for SPECT myocardial perfusion imaging (MPI) [33]. An example of SPECT ^123^I-*m*IBG images is shown in Figure 4. Furthermore, comparison of cardiac SPECT ^123^I-*m*IBG images with MPI can also be useful in specific cardiac pathologies such as ischemic heart disease. More recently, the introduction of dedicated cardiac cameras, equipped with solid-state Cadmium–Zinc–Telluride (CZT) detectors characterized by a higher photon sensitivity and spatial resolution compared to standard gamma cameras allow repeated assessment of cardiac innervation with lower radiation exposure [34]. Furthermore, these CZT detectors allow evaluating myocardial innervation and perfusion in a single session, i.e., dual isotope single acquisition imaging.

### 2.4. Standardization

Essential for large scale implementation of cardiac ^123^I-*m*IBG imaging is adequate reproducibility, standardization and validation. Therefore, based on scientific data a proposal for standardization of cardiac ^123^I-*m*IBG imaging has been published by Flotats et al. [27]. Table 1 summarized typical acquisition conditions for both planar and SPECT cardiac imaging. Furthermore, most cardiac ^123^I-*m*IBG imaging data is acquired from single center experiences and do not necessarily allow extrapolation to other institutions. One of the most contributing factors of variation of H/M outcome is difference in collimator and gamma camera combination. ^123^I high-energy photons (i.e.,1.4% 529 keV) causes scatter and collimator septal penetration. This degrades not only the image quality, but has also a significant effect on the H/M and ^123^I-*m*IBG WO calculation [35]. Therefore, a phantom-based correction method for different collimator and gamma camera use has been developed by Nakajima et al. [36,37]. This cross-calibration of H/M not only enables a better comparison between institutions, but also unifies H/M among various institutions in multicentre studies, which is important for identifying appropriate thresholds of H/M for differentiating high- and low-risk patients.

### 2.5. Challenges

Although cardiac ^123^I-*m*IBG imaging is feasible in many cardiac diseases, it can be challenging in subjects with severe impaired cardiac sympathetic innervation. Due to (very) low myocardial ^123^I-*m*IBG uptake, the anatomic borders of LV are difficult to recognize. In addition, in subjects with severe HF the LV is often dilated. Combined, this may hamper correct placement of the myocardial ROI and may therefore have an impact on the calculated H/M ratio. ^123^I-*m*IBG SPECT combined with CT (e.g., low dose CT for attenuation correction purposes only) may overcome these issues.

## 3. Cardiac ^123^I-*m*IBG Imaging in CHF

Increased cardiac sympathetic innervation is reflected by a decreased late H/M and increased WO. Both parameters have been shown to be important predictors of events in many cardiac diseases including atrial fibrillation, hypertrophic cardiomyopathy and chemotherapy induced cardiac toxicity [38,39,40]. However, since its introduction cardiac ^123^I-*m*IBG imaging has extensively been studied in ischemic and non-ischemic HF with reduced left ventricular function (HFREF). To date, there is limited information regarding cardiac sympathetic innervation in HF with preserved LVF (HFPEF) [40]. Therefore, the following paragraphs will discuss the role of cardiac ^123^I-*m*IBG imaging in CHF with HFREF only. At the end, we will discuss the role of cardiac ^123^I-*m*IBG imaging in a special type of HF, takotsubo cardiomyopathy (TCM).

### 3.1. Alteration of Cardiac Sympathetic Activity by Medical Heart Failure Therapy

In the past decades, the cornerstone of medical heart failure therapy is treatment with β-blockers and ACE-I/ARB. Although this medical therapy has a favorable effect on the LVEF and prognosis, it may also have an impact on cardiac sympathetic activity. β-blockers are thought to reduce the detrimental effects of NE stimulation in CHF [41]. A small randomized, multicenter study by Cohen-Solal et al. evaluated the effect of carvedilol on cardiac sympathetic activity in 64 CHF patients [42]. The authors concluded that benefits of carvedilol on resting hemodynamics appear to be associated with a partial recovery of cardiac sympathetic activity. Furthermore, it has been shown that ACE-I improves neuronal function with increased cardiac ^123^I-*m*IBG uptake [43,44]. This may be the result of direct improvement of NE uptake by reducing angiotensin II concentration. It has been reported that angiotensin II prevents re-uptake of NE via the NET [44]. This may lead to increased NE levels in the synaptic cleft and consequently increased stimulation of the post-synaptic β-AR of the myocytes. However, as described previously, long-term NE excess has detrimental effects with down-regulation of post-synaptic β-AR. Furthermore ACE-I is known to improve the hemodynamics. This systemic effect may indirectly result in reduced NE release and normalization of NE uptake by NET. Recently, angiotensin-receptor neprilysine-inhibitor (ARNI) and sodium-glucose cotransporter-2 (SGLT-2) inhibitors have been added to the medical treatment for CHF with impressive effects on both morbidity and mortality [45,46,47]. However, the effect of ARNI and SGLT-2 inhibition on cardiac sympathetic activity in CHF is still unknown and needs further investigation. Although heart failure therapy may alter the prognosis of HF there is still a residual risk of HF progression, ventricular arrhythmia and SCD. In the next paragraphs we will discuss the use of cardiac ^123^I-*m*IBG imaging to access this residual risk despite optimal medical HF therapy.

### 3.2. Cardiac ^123^I-mIBG Imaging as a Predictor of Morbidity and Mortality in CHF

Since the first study of ^123^I-*m*IBG assessed cardiac sympathetic innervation in CHF by Merlet et al. [47], a large number of small prospective and retrospective studies have examined the relevance of cardiac sympathetic innervation assessed with cardiac ^123^I-*m*IBG imaging as a predictor of cardiac events including HF progression, fatal arrhythmia and cardiac death [31,48,49,50]. CHF patients with increased cardiac sympathetic innervation (i.e., reduced late H/M and increased ^123^I-*m*IBG WO) had a worse prognosis compared with those with relatively preserved cardiac sympathetic innervation. These findings were confirmed in the multicentre ADMIRE-HF (ADreView Myocardial Imaging for Risk Evaluation in Heart Failure) study, that prospectively evaluated the prognostic significance of cardiac ^123^I-*m*IBG imaging in 961 stable CHF patients on OMT with New York Heart Association (NYHA) class II or III and a LVEF ≤ 35% [22]. A predefined late H/M cut-off value of 1.6 using a LEHR collimator was, independent from commonly used markers (i.e., BNP and LVEF), a predictor of the composite endpoint and of each individual component of the composite endpoint: occurrence of HF progression, lethal ventricular tachycardia (VT), or cardiac death. The risk of a cardiac event was significantly higher in patients with a late H/M < 1.6 compared to patients with late H/M > 1.6, with a 2-year event rate of 37% vs. 15% (*p* < 0.001). The 2-year risk of death was significantly higher in patients with late H/M < 1.6 compared to patients with late H/M > 1.6 with an all-cause mortality rate of 16.1% vs. 3.0% (*p* < 0.001) and with cardiac mortality rate of 11.2% vs. 1.8% (*p* = 0.001). In addition, when late H/M treated as a continuous variable, there was a progressive decline in both all-cause and cardiac mortality from 20% for late H/M < 1.1 to none for late H/M ≥ 1.8. Recently the long-term follow-up data of the ADMIRE-HF study with a median follow-up of 62.7 months showed similar results with a significantly higher risk of death in patients with late H/M < 1.6 compared to patients with late H/M > 1.6 with an all-cause mortality rate of 38.4% vs. 20.9% (*p* < 0.001) and with cardiac mortality rate of 16.8% vs. 4.5% (*p* < 0.001) [51]. Since the ADMIRE-HF study a late H/M cut-off value of 1.6 became accepted to discriminate low and high-risk patients. However, this cut-off point is based on LEHR collimator use only. For institutions using other collimator types than LEHR, this cut-off value should be corrected using the previous described cross-calibration phantom [36,37]. For example, for intuitions using medium energy general purpose (MEGP) collimator a H/M should be interpreted as 1.96.

A pooled analyses of independent studies using original individual patient and image data, confirmed the results of the ADMIRE-HF study that cardiac ^123^I-*m*IBG imaging has long-term prognostic value in CHF [52,53]. Interestingly, a meta-analysis of 6 studies including 636 CHF patients showed that late H/M is not only useful as a dichotomous predictor of prognosis (i.e., high vs. low risk), but also has prognostic implication over the full range of the outcome value for all event categories except ventricular arrhythmias [52]. This finding showed a clear linear relationship between the amount of myocardial dysinnervation and overall prognosis in CHF with HFREF.

### 3.3. Cardiac ^123^I-mIBG Imaging as a Predictor for Arrhythmia and ICD Therapy in CHF

Despite medical therapeutic improvements in recent decades, the prognosis of CHF remains inauspicious partly due to fatal arrhythmias and SCD [54]. However, since the introduction of ICDs the overall survival of CHF patients has improved significant [55,56,57]. ICD implantation is indicated in survivors of sustained VT of ventricular fibrillation (VF) (i.e., secondary prevention), but also in selected patients without prior ventricular arrhythmia (i.e., primary prevention) ICD implantation is indicated. Based on large randomized studies, current ESC guidelines recommend ICD implantation for primary prevention in symptomatic stable CHF subjects with NYHA class ≥ 2, LVEF ≤ 35% and under OMT [46].

However, despite the selection criteria for ICD implantation (primary or secondary prevention) it has been reported that 65% of the patients never received appropriate ICD therapy in the first 3 years after implantation [58]. Furthermore, the SCD-HeFT (Sudden Cardiac Death in Heart Failure Trial) study showed that 1 year after implantation the annual number of appropriate ICD therapy was only 5.1% rising to 21% 5 years after implantation [57]. Therefore, more precise patient tailored risk-stratification is needed in order to achieve a more (cost)effective management of CHF.

Although the exact pathophysiology of ventricular arrhythmias is multifactorial, it has been recognized that increased myocardial sympathetic innervation is an important factor in the origin of ventricular arrhythmias in patients with HFREF, especially in ischemic HF [59]. In these patients ventricular arrhythmias develop in myocardial areas with slow conduction in relation to enhanced automaticity, triggered automaticity, and re-entrant mechanisms [60]. In addition, non-uniform denervated myocardium in infarct zone can be hypersensitive to released NE in the synaptic cleft. Especially the border zone of infarct areas with viable myocardium is predisposed to develop re-entrant circuits. This mechanism is most likely triggered by the fact that sympathetic nerve fibres are more susceptible to ischemia than myocytes, thereby causing a disbalance between still viable but partly denervated and normal myocardium [61].

Unlike the clear linear relation between overall prognosis and cardiac sympathetic innervation in CHF with HFREF [52], the exact relation between fatal arrhythmia (i.e., sustained VT or VF) and cardiac sympathetic innervation remains unclear. Some smaller single center studies suggested an association between increased cardiac sympathetic innervation and ventricular arrhythmia or appropriate ICD therapy [62,63,64]. For example, a prospective study by Boogers et al. including 116 CHF patients, eligible for ICD implantation for both primary and secondary prevention of SCD, ^123^I-*m*IBG SPECT, as a dichotomous variable, was shown to be an independent predictor of appropriate ICD therapy (i.e., anti-tachypacing or shock) and cardiac death [63]. A prospectively selected median summed defect score (SDS) cut-off of 26 was used. The cumulative incidence of appropriate ICD therapy during 3-year follow-up was significantly higher in patients with a relatively large ^123^I-*m*IBG SPECT defect (SDS > 26) compared to patients with a small ^123^I-*m*IBG SPECT defect (SDS ≤ 26) (52% vs. 5%, *p* < 0.01). Another small study including 27 CHF patients referred for ICD implantation for primary prevention of SCD only, showed that patients with fatal arrhythmia and SCD had lower late H/M (1.54 vs. 1.96, *p* < 0.001) and higher ^123^I-*m*IBG SPECT SDS (37.0 vs. 25.5, *p* = 0.002) compared to those without fatal arrhythmia and SCD [62]. The PAREPET (Prediction of ARrhythmic Events with Positron Emission Tomography) study showed similar result for PET imaging. In patients with ischemic HF eligible for implantable cardioverter defibrillator (ICD) for primary prevention of SCD the extent of ^11^C-HED assesses impaired cardiac sympathetic innervation was a predictor of SCD independently of LVEF, infarct volume, cardiac symptoms, and brain natriuretic peptide (BNP) plasma levels [65]. Interestingly, a recent study including 94 patients with ischemic HF referred for ICD implantation for primary or secondary prevention of SCD showed that ^123^I-*m*IBG-derived parameters could not predict appropriate ICD therapy in patients with an ICD for primary prevention of SCD [66]. Although patients with appropriate ICD therapy (i.e., anti-tachypacing or shock) for secondary prevention of SCD had a larger innervation/perfusion mismatch, but no significant difference in early and late H/M. So, except from some small studies, a linear relation between ^123^I-*m*IBG scintigraphy findings (i.e., late H/M, ^123^I-*m*IBG WO and ^123^I-*m*IBG SPECT SDS) and the occurrence of potentially fatal arrhythmia or appropriate ICD therapy is lacking [52]. An explanation could be the heterogeneity of the study population including ICD implantation for primary vs. secondary prevention of SCD and ischemic vs. non-ischemic HF.

**Figure 5:**
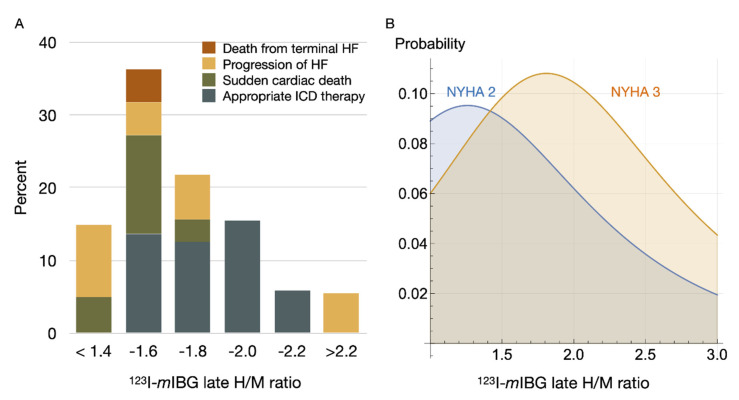
^123^I-*m*IBG H/M and breakdown of serious events (**A**); death from terminal heart failure (HF), progression of HF, sudden cardiac death, and appropriate ICD therapy [67]. The right panel (**B**) shows machine-learning-based simulation of probability of fatal arrhythmic death. In this simulation a model was created based on patients with documented 2-year outcomes of CHF using 13 variables including age, gender, NYHA functional class, left ventricular ejection fraction and planar ^123^I-*m*IBG late H/M ratio [71]. The bell-shape appearance of serious arrhythmic events as observed in clinical studies is replicated by simulation models as well.

Recently, a multicentre study including 135 stable CHF subjects (age 64.5 ± 9.3 years, 79% male, LVEF 25 ± 6%) referred for ICD implantation for primary prevention only, showed a “bell-shape” relation between ^123^I-*m*IBG scintigraphy findings (using standardized H/M) and the occurrence of appropriate ICD therapy (i.e., anti-tachypacing or shock) [67]. Patients with intermediate late H/M (range 1.40–2.10) were more likely to receive appropriate ICD therapy compared to patients with low and high late H/M (Figure 5A). These findings are in line with previous findings by Agostini et al. [31]. Arrhythmia occurred in CHF patients with an intermediate late H/M between 1.46 and 2.17. Similar results were shown for ^123^I-*m*IBG SPECT imaging [68]. In 471 ischemic CHF patients, those with intermediate defects on ^123^I-*m*IBG SPECT SDS appeared to be at the highest risk for arrhythmic events (i.e., sustained VT, resuscitated cardiac arrest, appropriate ICD therapy). Therefore, the authors concluded that the presumption of a linear increase in risk of an arrhythmic event with increasing ^123^I-*m*IBG SPECT defects may not be correct. More recently, a new wall-level based scoring method was suggested for analyzing innervation/perfusion mismatch in ischemic HF [69]. This visual wall-level based scoring method identified highest risk for fatal arrhythmia (i.e., sustained VT, resuscitated cardiac arrest, appropriate ICD therapy or SCD) in ischemic HF patient with intermediate levels of innervation/perfusion mismatches. The results of the previous studies [31,68,69,70] with a ‘‘bell-shaped’’ curve for ^123^I-*m*IBG-derived parameters (i.e., late H/M or ^123^I-*m*IBG SPECT SDS) in relation to ventricular arrhythmia or appropriated ICD therapy underline the previous described hypothesis of the occurrence of ventricular arrhythmias in ischemic HF patients. More importantly, these studies suggest that cardiac ^123^I-*m*IBG imaging could play a role in patient selection for an expensive device therapy such as ICD implantation.

Finally, cardiac ^123^I-*m*IBG scintigraphy-guided selection of candidates for ICD implantation seems to be cost-effective [72]. In a cost-effectiveness model, cardiac ^123^I-*m*IBG screening in CHF patients was associated with a reduction in ICD implantation by 21%, resulting in a number needed to screen to prevent 1 ICD implantation of 5. Consequently, compared to no cardiac ^123^I-*m*IBG screening costs per patient were reduced by USD5500 and USD13,431 over 2 and 10 years, respectively. Screening with cardiac ^123^I-*m*IBG imaging resulted in losses of 0.001 and 0.040 life years over 2 and 10 years, respectively. Although larger studies are necessary to define the exact role of cardiac ^123^I-*m*IBG imaging in patient selection for ICD implantation, these findings are encouraging in better discriminating those patients who may benefit from those who do not benefit from ICD implantation.

### 3.4. Cardiac ^123^I-mIBG Imaging as Predictor of CRT Response

Cardiac resynchronization therapy (CRT) is a disease modifying therapy. In selected CHF patients (left bundle branch block (LBBB), QRS duration ≥ 150 msec, LVEF ≤ 35% and NYHA class ≥ 2) CRT reduces morbidity and mortality as a result of reverse remodeling (i.e., improvement of LVEF) [46,73]. Despite these guidelines recommended selection criteria, only one-third of these CHF patients does not benefit from this invasive and expensive therapy. A recent review, including 9 studies with a total of 225 CHF patients, evaluated CRT and ^123^I-*m*IBG assessed cardiac sympathetic innervation [74]. As a uniform definition of response criteria for CRT is lacking, most studies used different criteria for CRT response. However, all available studies showed positive changes in cardiac sympathetic innervation in the responders to CRT. Furthermore, cardiac ^123^I-*m*IBG imaging seems to be promising in identifying CHF patients who do not benefit from CRT. This was confirmed by the BETTER-HF study including 121 CHF patients. This study showed that baseline late H/M was an independent predictor of CRT response defined as LV remodelling with 15% reduction in left ventricular end systolic volume (LVESV) (regression coefficient 2.906, [0.293–3.903], *p* = 0.029) [75]. Furthermore, cardiac sympathetic innervation was improved only in those patients who responded to CRT and these positive changes were correlated with improvement in functional capacity. Although these data are promising, extrapolation to other institutions is hampered by the lack of uniform CRT response criteria and differences in collimator use. To overcome issues of different collimator use, recently a multicentre study evaluated ^123^I-*m*IBG assessed cardiac innervation in relation to response to CRT by using standardized H/M [76]. In total 78 stable CHF subjects (LBBB, QRS duration ≥ 150 msec, LVEF ≤ 35% and NYHA class ≥ 2) referred for CRT implantation were enrolled. The results showed that early and late H/M were independent predictors of CRT response (i.e., improvement of LVEF). Therefore, cardiac ^123^I-*m*IBG imaging could be used as a tool to select subjects that might benefit from CRT.

## 4. Risk Stratification Using Cardiac ^123^I-*m*IBG Imaging

To enhance the utility of risk markers for CHF, a large number of multivariate risk models have been developed in the past decades [77,78,79,80]. Although these models often use readily clinical available data (i.e., age, gender, NT-proBNP, NYHA class, LVEF), the use of these risk models in clinical practice remains limited. In Japan, where cardiac ^123^I-*m*IBG imaging is already recommended in the national heart failure guidelines [81], a risk model was developed for predicting 5-year cardiac mortality in CHF patients using a pooled database [82]. Parameters used for this model included age, gender, NYHA class and LVEF. Interestingly, by adding late H/M to the model the net reclassification improvement analysis for all subjects was 13.8% (*p* < 0.0001). This addition was most effective in the downward reclassification of low-risk patients. Furthermore, mortality risk charts for CHF patients have been developed using the following parameters: age, NYHA class, LVEF, and late H/M. These risk charts are based on 2- and 5-year risk models using a pooled database including 1388 CHF patients [83].

Recently, Nakajima et al. have developed a machine learning risk model for predicting 2 years risk of fatal arrhythmia and HF death in CHF patients [71]. In total 13 parameters were used including age, gender, NYHA class, LVEF and late H/M. The probability of HF death is inversely proportional to late H/M with a significantly increase in probability of HF death as late H/M decreased. However, for fatal arrhythmia the probability was maximal when late H/M was intermediate, especially in NYHA class II and III (Figure 5B). This is in line with observations in previously described studies showing a so called ‘‘bell-shaped’’ curve of fatal arrhythmia in relation to ^123^I-*m*IBG-derived parameters [67,68,69,70].

## 5. Cardiac ^123^I-*m*IBG Imaging in Takotsubo Cardiomyopathy

Takotsubo cardiomyopathy (TCM), a special type of HF, is characterized by acute chest pain and is associated with electrocardiographic (ECG) changes, elevated troponins and transient LV dysfunction with apical and mid ventricular dyskinesia in the absence of coronary artery disease which mimics an acute coronary syndrome (ACS) [84]. The onset of TCM is commonly triggered by exposure to acute emotional of physical stress. Although the precise pathophysiology of this syndrome has not been completely elucidated, considerable evidence points to epinephrine as an important factor in the pathophysiology. Exposure to high levels of epinephrine may change the intracellular signalling in the myocytes with shifts from positively inotropic G2 coupling to negative inotropic G-inhibitor (Gi) coupling of the β_2_ adenoreceptors (β_2_AR) [85]. The mechanism of regional wall motion difference between apex and base is probable due to a greater proportion of β_2_AR relative to β_1_AR in the apex compared to the base [86]. Paur et al. showed that this higher β_2_AR:β_1_AR ratio in the apex makes this part of the LV more vulnerable to excessive epinephrine stimulation [85]. This could explain the decreased apical and preserved basal wall motion in the acute phase of TCM.

**Figure 6:**
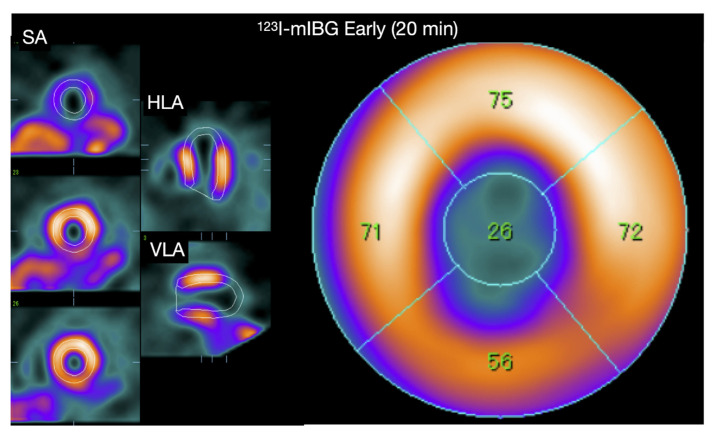
A 60-year-old Japanese woman diagnosed with Takotsubo syndrome. The ^123^I-*m*IBG early SPECT show a clear defect in the apical region, and the late image also showed a similar defect (figure not shown). The early and late planar H/M ratio of this patient are shown in Figure 2, showing preserved ^123^I-*m*IBG uptake globally despite the severe apical defect.

Although TCM is associated with increase epinephrine levels, cardiac ^123^I-*m*IBG imaging shows decrease apical ^123^I-*m*IBG uptake in the sub-acute phase of TCM (Figure 2) [87]. This is also seen in cardiac ^123^I-*m*IBG SPECT imaging (Figure 6) [88]. It has been demonstrated that high levels of epinephrine inhibit the NE uptake by NET [89]. Therefore, the reduced ^123^I-*m*IBG uptake (i.e., NE) via NET could be explained as an indirect effect of high epinephrine levels. It seems that the reduced uptake of ^123^I-*m*IBG correlates with the impaired LV segments. Recently, Matsuura et al. evaluated the relationship between ^123^I-*m*IBG assessed cardiac sympathetic innervation and LVF improvement and the correlation with clinical outcomes in TCM [90]. In total 90 patients with TCM were enrolled and were divided into 2 groups of LVF improvement: <1 month (E) and >1 month (L) The L group was characterized by high catecholamine plasma levels and lower late H/M (2.09 ± 0.45 vs. 2.45 ± 0.44, *p* = 0.01) with higher ^123^I-*m*IBG WO (33.9% ± 13.8% vs. 26.4% ± 10.2%, *p* = 0.02) compared to the E group. The in-hospital complications were higher in the L group compared to the E group (56% vs. 33%, *p* = 0.03) including HF (45% vs. 23%, *p* = 0.03) and in-hospital death (8% vs. 0%, *p* = 0.03). The authors concluded that in TCM, increased cardiac sympathetic activity was observed in patients with delayed LVF recovery, which was associated with adverse in-hospital outcomes. Akashi et al. evaluated 8 patients with TCM using both planar and SPECT cardiac ^123^I-*m*IBG imaging [91]. After 3 months of follow-up the impaired late H/M was increased compared to baseline (1.89 ± 0.25 vs. 2.13 ± 0.24, *p* < 0.05). In addition, the ^123^I-*m*IBG WO significantly improved compared to baseline (39.1% ± 10.3% vs. 25.4% ± 6.3%, *p* < 0.05). The authors concluded that this transient regional impaired cardiac sympathetic innervation may causes transient neurogenic myocardial stunning in TCM. Although the late H/M did not completely recover after 3 months, it has been demonstrated by Owa et al. that 1 year after the onset of TCM late H/M completely recovers [92]. Of interest is that despite normalization of LVF and epinephrine plasma levels after a few weeks of onset of TCM, cardiac sympathetic activity takes longer to recover in some patients. The mechanism of this prolonged impaired ^123^I-*m*IBG uptake has not been elucidated yet. It has been suggested that the relatively high density and increased sensitivity of apical of β_2_AR to epinephrine causes a prolonged effect of downregulation of β_2_AR and impaired re-uptake by NET [93]. This leads to relatively high levels of epinephrine and NE in the synaptic cleft. As a consequence, the high levels cause a slow recovery of apical β_2_AR and NET compared to the basal located β_2_AR and NET. In addition, this slow recovery of cardiac sympathetic innervation may identify those patients that are more at risk for a recurrent episode of TCM.

## 6. Clinical Acceptation of Cardiac ^123^I-*m*IBG Imaging

Despite the numerous studies showing changes of ^123^I-*m*IBG assessed cardiac sympathetic activity as a measure of response to pharmaceutical or device therapy [35,37,38,59,67] and the enormous number of outcome studies demonstrating the prognostic significance of ^123^I-*m*IBG assessed cardiac sympathetic activity in HF [14,45,86] most cardiologists are not convinced of the additional value and relevance of this non-invasive imaging technique in routine clinical practice. Although the difference between a predicted 2% and 10% annual mortality risk may be statistically significant, if this reflects the already treated underlying, the information of annual mortality risk will unlikely change how the cardiologist treats this patient. Even for the risk for SCD in a CHF subject eligible for an ICD, almost all cardiologists would adhere to the guidelines [46] even if cardiac ^123^I-*m*IBG imaging suggested the patient’s true arrhythmic event risk was extremely low [94].

Currently, there are no randomized clinical trials that have evaluated cardiac ^123^I-*m*IBG-guided therapy improves outcomes in CHF subject. Without such data, cardiologists have little incentive to order cardiac ^123^I-*m*IBG scintigraphy for clinical decision making, Eventual approval of a cardiac PET agent capable of quantifying sympathetic innervation will probably make the challenge of convincing the cardiologist to use cardiac ^123^I-*m*IBG scintigraphy for clinical decision making even more daunting. In contrast to locations where ^123^I-*m*IBG is relatively inexpensive and can be used as a binary diagnostic test agent, it seems unlikely there will be significant growth in cardiac ^123^I-*m*IBG imaging in the foreseeable future. However, given the increasing medical costs associated with CHF, a better selection of subjects for expensive device therapy, such as ICD and CRT, is mandatory. The current selection criteria fail to make a proper selection of patients that benefit from these devices [46]. If there is a potential clinical role for cardiac ^123^I-*m*IBG imaging in CHF, it will be in guiding the selection of these CHF subjects. Although currently available data show promising result for cardiac ^123^I-*m*IBG imaging, none of these studies was designed to demonstrate that ^123^I-*m*IBG-guided findings can be used to improve patient outcomes.

## 7. Conclusions

Cardiac ^123^I-*m*IBG imaging is a non-invasively, widely available imaging technique and has been established to evaluate the prognosis in CHF. Standardization, especially among various gamma camera–collimator combinations is important for identifying appropriate thresholds for adequate risk stratification and extrapolation of data to other institutions. Most importantly, in contrast to the linear relationship between ^123^I-*m*IBG-derived parameters and the overall prognosis in CHF, there seems a “bell-shape” curve for ^123^I-*m*IBG-derived parameters in relation to fatal arrhythmias. These new insights could be helpful, especially in the optimization of patient selection for expensive device implantation such as ICD and CRT.

## Figures and Tables

**Table 1:** Comparison of the neuronal handling of radiotracers for imaging cardiac sympathetic innervation.

Tracer	Imaging	Affinity for NET	Vesicular Storage	Vesicular Leakage	Sensitivity to MAO/COMT	Neuronal Membrane Leakage	Cause of Imaging Defect/Uptake reduction
**^123^I-*m*IBG**	Planar and SPECT	+++	++	+	Minimal	+	↓ Nerve density ↓ NET ↑ Sympathetic activity ↑ NE (competitive inhibiton for NET)
**^11^C-EPI**	PET	++	+++	Minimal	+	Minimal	↓ Nerve density ↓ NET ↑ Sympathetic activity ↑ NE (competitive inhibiton for NET) ↑ MAO activity ↓ VMAT2 ↓ Vesicular storage
**^11^C-PHEN**	PET	+	+	++	++	++	↓ Nerve density ↓ NET ↑ Sympathetic activity ↑ NE (competitive inhibiton for NET) ↑ MAO activity ↓ VMAT2 ↓ Vesicular storage
**^18^F-Flubrobenguane**	PET	+++	++	+	Minimal	+	↓ Nerve density ↓ NET ↑ Sympathetic activity ↑ NE (competitive inhibiton for NET)

Data presented in this table are relative. NET affinity data from [12,13,14],vesicular transport data from [12,13], vesicle leakage data from [15,16], neuronal membrane leakage data from [8,13,14,16,17]. Adapted with permission from Zelt et al. [18]. COMT: catecholamine-O-methyl-transferase; ^11^C-EPI: epinephrine, F-Flubrobenguane: N-[3-Bromo-4-(3-^18^F-fluoro-propoxy)-benzyl]-guanidine, ^11^C-HED: *meta*-hydroxyephedrine, MAO: monoamine oxidase, ^123^I-*m*IBG: *meta*-idobenzylguanidine, NE: norepinephrine, PET: positron emission tomography, ^11^C-PHEN: phenylephrine, SPECT: single-photon emission computed tomography, VMAT2: vesicular monamine transporter 2.

**Table 2:** Recommended cardiac ^123^I-*m*IBG imaging acquisition conditions. * standard dose of ^123^I-*m*IBG varies among countries; 111 MBq in Japan, 185 MBq in Europe, and 370 MBq in the USA. ** Total acquisition time 20–30 min with anger camera and 10 min with cardiac CZT camera.

Cardiac ^123^I-*m*IBG imaging	
Administration dosis of ^123^I-*m*IBG	111–370 MBq *
Timing of acquisition p.i.	15–30 min (early)
	3–4 h (late)
Planar imaging	128 × 128 of 256 × 256 matrix
	5–10 min
	LE of ME collimators (standarization recommended)
SPECT imaging	64 × 64 matrix
	3–6 degree step, 30 s per projection **
	180 or 360 degree rotation

## Data Availability

Data sharing not applicable.

## Abbreviations

^123^I-*m*IBG	^123^I-*meta*-iodobenzylguanidine
ACE-I	angiotensin-converting enzyme inhibitors
ACS	acute coronary syndrome
ARB	angiotensin receptor blockers
ARNI	angiotensin-receptor neprilysine-inhibitor
BNP	brain natriuretic peptide
β-AR	β-adrenergic receptors
CHF	chronic heart failure
CZT	Cadmium–Zinc–Telluride
FBBG	Flubrobenguane
ICD	implantable cardioverter defibrillator
HED	hydroxyephedrine
HFPEF	heart failure with preserved ejection fraction
HFREF	heart failure with reduced ejection fraction
HRV	heart rate variability
H/M	heart-to-mediastinum ratio
LBBB	left bundle branch block
LVEF	left ventricular ejection fraction
LVESV	left ventricular end systolic volume
MAO	monoamine oxidase
MPI	myocardial perfusion imaging
NE	norepinephrine
NET	norepinephrine transporter
NYHA	New York heart association
OMT	optimal medical therapy
ROI	region-of-interest
SCD	sudden cardiac death
SDS	summed defect score
SGLT2 inhibitor	sodium-glucose cotransporter-2 inhibitor
SPECT	single-photon emission tomography
TCM	takotsubo cardiomyopathy
WO	washout
